# Convalescent-Phase Sera and Vaccine-Elicited Antibodies Largely Maintain Neutralizing Titer against Global SARS-CoV-2 Variant Spikes

**DOI:** 10.1128/mBio.00696-21

**Published:** 2021-06-01

**Authors:** Takuya Tada, Belinda M. Dcosta, Marie I. Samanovic, Ramin S. Herati, Amber Cornelius, Hao Zhou, Ada Vaill, Wes Kazmierski, Mark J. Mulligan, Nathaniel R. Landau

**Affiliations:** a Department of Microbiology, NYU Grossman School of Medicine, New York, New York, USA; b NYU Langone Vaccine Center, NYU Grossman School of Medicine, New York, New York, USA; c Department of Medicine, NYU Grossman School of Medicine, New York, New York, USA; d Biohaven Pharmaceuticals, Inc., New Haven, Connecticut, USA; Columbia University/HHMI

**Keywords:** SARS-CoV-2, neutralization, B.1.1.7, B.1.351, B.1.1.248, COH.20G/677H, 20A.EU2, mink cluster 5, spike protein, Pfizer BNT162b2, REGN10933, REGN10987

## Abstract

The increasing prevalence of severe acute respiratory syndrome coronavirus 2 (SARS-CoV-2) variants with spike protein mutations raises concerns that antibodies elicited by natural infection or vaccination and therapeutic monoclonal antibodies will become less effective. We show that convalescent-phase sera neutralize pseudotyped viruses with the B.1.1.7, B.1.351, B.1.1.248, COH.20G/677H, 20A.EU2, and mink cluster 5 spike proteins with only a minor loss in titer. Similarly, antibodies elicited by Pfizer BNT162b2 vaccination neutralized B.1.351 and B.1.1.248 with only a 3-fold decrease in titer, an effect attributable to E484K. Analysis of the Regeneron monoclonal antibodies REGN10933 and REGN10987 showed that REGN10933 has lost neutralizing activity against the B.1.351 and B.1.1.248 pseudotyped viruses, and the cocktail is 9- to 15-fold decreased in titer. These findings suggest that antibodies elicited by natural infection and by the Pfizer vaccine will maintain protection against the B.1.1.7, B.1.351, and B.1.1.248 variants but that monoclonal antibody therapy may be less effective for patients infected with B.1.351 or B.1.1.248 SARS-CoV-2.

## INTRODUCTION

Since the zoonotic transfer of severe acute respiratory syndrome coronavirus 2 (SARS-CoV-2) to humans at the end of 2019, the virus has rapidly mutated to adapt to its new host. Such adaptations are a feature of viral zoonoses in which selective pressure drives viral proteins to be optimized for interaction with the host cell proteins of the new species. In addition, the amino acid sequences of virus proteins are selected to escape the humoral and cellular adaptive immune responses of the new host, which recognizes a different set of epitopes. While all of the viral genes are subject to evolutionary pressure, the viral envelope glycoprotein is selected both for optimal interaction with its cell surface receptor and for escape from neutralizing antibodies, driving its rapid evolution.

Following the isolation of Wuhan-Hu1 in December 2019, SARS-CoV-2 has continued to evolve as it adapts to it’s new host. A variant with a D614G mutation in the spike protein was identified in January 2020, and by May, the variant had become the predominant strain worldwide, with a prevalence of >97% (1). The mutation, which is located near the S1:S2 processing site, reduces S1 subunit shedding from virions, resulting in increased infectivity and higher virus loads (2–4). Additional variants with increased transmissibility were subsequently identified, each containing D614G. The B.1.1.7 lineage (VOC-202012/01) variant identified in patients in the United Kingdom (5–7) encodes a spike protein with 8 mutations in addition to D614G (Δ69–70, Y144Del, N501Y, A570D, P681H, T716I, S982A, and D1118H). N501Y is one of six ACE2 contact residues and has been shown to increase the affinity for ACE2 by forming a hydrogen bond with Y41 (8, 9), the Δ69–70 deletion in the N-terminal domain (NTD) is found in multiple independent lineages (10), and P681H lies adjacent to the furin cleavage site (11), suggesting a role in spike protein processing.

The B.1.351 lineage variant identified in South Africa that has become the predominant genotype in that population is more heavily mutated than B.1.1.7, with 9 mutations (L18F, D80A, D215G, L242-244del, R246I, K417N, E484K, N501Y, and A701V), 3 of which (K417N, E484K, and N501Y) are in the receptor binding domain (RBD) (12). E484K, like N501Y, lies in the receptor binding motif (RBM) that directly contacts specific ACE2 residues (13). K417N, while not contributing to ACE2 binding, is an epitope for neutralizing antibodies, as is E484K, and thus may have been selected for evasion of the humoral response. Based on phylogenetic tree branch length, it has been suggested that the variant arose through prolonged virus replication in an immunocompromised individual (10). The P1 Brazilian variant B.1.1.248 is similar to the South Africa variant, with four additional mutations in the NTD and two mutations in S2 (14). Additional variants include 20A.EU2, which was identified in Spain (15) and later found elsewhere in Europe, and COH.20G/677H, which was identified in Columbus, OH (16). The COH.20G/677H spike protein contains D614G, N501Y, and Q677H but lacks the mutations present in B.1.1.7 and B.1.351, suggesting an independent origin (16). In addition, a variant found in domesticated minks in Denmark, designated mink cluster 5 (Δ69–70/Y453F/I692V/M1229I), has the potential for transfer to humans (17).

The emergence of SARS-CoV-2 variants with mutated spike proteins raises concerns regarding a loss of protection from reinfection and decreased protection by vaccination (13, 18–30). The Pfizer-BioNTech BNT162b2 and Moderna mRNA-1273 vaccines are lipid-nanoparticle-formulated, modified nucleoside mRNAs that encode a trimerized spike protein (31, 32), and both are currently based on the parental SARS-CoV-2 amino acid sequence. SARS-CoV-2 variant spike proteins also pose a challenge for monoclonal antibody (mAb) therapy for coronavirus disease 2019 (COVID-19) (33, 34). The Regeneron Pharmaceuticals REGN-COV2 therapy is a cocktail of two recombinant monoclonal antibodies consisting of REGN10933 (casirivimab) and REGN10987 (imdevimab) (35, 36), while the Eli Lilly therapy is based on a single antibody, LY-CoV016 (37). The antibodies bind epitopes in the RBD of the Wuhan-Hu1 spike protein. Because monoclonal antibodies bind a single epitope on the spike protein, they are particularly susceptible to escape, rendering the therapy ineffective. Recent findings have demonstrated partial escape of the B.1.351 variant and, to a lesser extent, B.1.1.7 from neutralization by the serum antibodies of convalescent patients and by antibodies elicited by the Pfizer-BioNTech BNT162b2 and Moderna mRNA-1273 mRNA vaccines that encode trimerized spike proteins. The decreased neutralizing titers against B.1.351 were largely the result of the E484K mutation, an amino acid residue that serves as a contact point for ACE2 (21, 23–30).

We report here on the ability of convalescent-phase sera, sera from individuals vaccinated with the Pfizer BNT162b2 vaccine, and the Regeneron monoclonal antibody cocktail to neutralize pseudotyped viruses with the variant spike proteins. The results showed that convalescent-phase serum maintained its neutralizing titers against the variants, with a <2-fold decrease in the neutralizing titer. Vaccine-elicited antibodies showed potent neutralization of pseudotyped virus with the B.1.1.7 spike protein, while pseudotyped viruses with the B.1.351 and B.1.1.248 spikes were neutralized with a 3- to 4-fold reduction in titer, which remained higher than those in the sera of naturally infected individuals. REGN10987 retains most of its activity against the variants, and REGN10933 failed to neutralize B.1.351, B.1.1.248, or mink cluster 5 pseudotyped viruses, an effect that mapped to E484K and K417N. The combination cocktail was decreased in neutralizing titers 9.1-fold against B.1.351, 15.7-fold against B.1.1.248, and 16.2-fold against mink cluster 5, raising concerns about the effectiveness of the cocktail treatment for individuals infected with the variant viruses. An analysis of the infectivity, thermostability, and ACE2 binding affinity showed that the variant spike proteins bound ACE2 with increased affinity and were more stable than the parental D614G. These findings suggest that antibodies elicited by infection with SARS-CoV-2 and by vaccination will protect against infection with currently circulating SARS-CoV-2 variants but raise concerns regarding monoclonal antibody therapies.

## RESULTS

**FIG 1:**
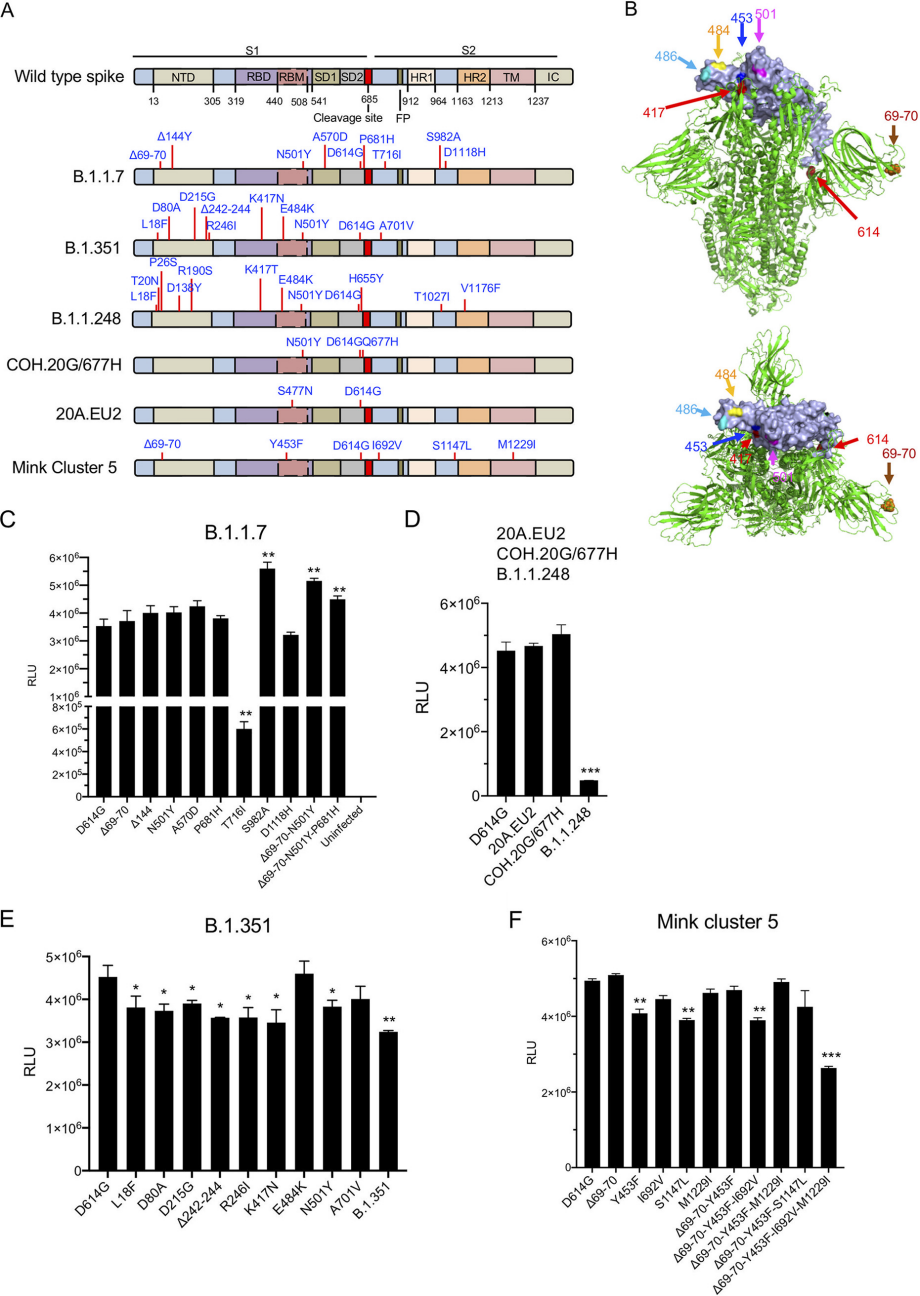
Infectivity of viruses pseudotyped by variant spike proteins. (A, top) Diagram showing the domain structure of the SARS-CoV-2 spike monomer. NTD, N-terminal domain; RBD, receptor binding domain; RBM, receptor binding motif; SD1 subdomain 1; SD2, subdomain 2; FP, fusion peptide; HR1, heptad repeat 1; HR2, heptad repeat 2; TM, transmembrane region; IC, intracellular domain. (Bottom) Diagrams indicating the locations of the mutations of the United Kingdom B.1.1.7; South Africa B.1.351; Brazilian B.1.1.248; Columbus, OH, COH.20G/677H; European 20A.EU2; and mink cluster 5 variant spikes. Expression vectors for the variant spike proteins or with the individual mutations were generated, each with the D614G mutation and deleted for the carboxy-terminal 19 amino acids. The vectors were used to produce pseudotyped lentiviral virions. The infectivity of the virions normalized for RT activity was tested on ACE2.293T cells. Luciferase activity was measured at 2 days postinfection. (B) Locations of key mutated amino acid residues in the spike protein. Side (top) and top (bottom) views of the SARS-CoV-2 spike protein prefusion structure with a single RBD (gray) are shown. Arrows indicate RBD amino acid residues 417, 453, 484, 486, and 501 and the Δ69–70 and D614G mutations. (C) Infectivity of viruses pseudotyped with the individual mutations of the B.1.1.7 spike protein or combinations thereof. RLU, relative luminescence units. (D) Infectivity of D614G; European 20A.EU2; Columbus, OH, COH.20G/677H; and Brazilian B.1.1.248 spike protein-pseudotyped viruses. (E) Infectivity of viruses pseudotyped with the South Africa B.1.351 variant spike protein and its individual mutations. (F) Infectivity of virus with the mink-associated variant spike protein individual mutations and combinations thereof. The experiments were repeated three times, with similar results.

The trimeric SARS-CoV-2 spike protein is synthesized as a full-length precursor polypeptide that is processed by cellular proteases into S1 and S2 subunits (Fig. 1A). S1, which mediates cell attachment, consists of an amino-terminal domain, the RBD, an RBM within the RBD that directly contacts ACE2, and subdomains 1 and 2. S2, which mediates membrane fusion, consists of a hydrophobic amino-terminal fusion peptide, heptad repeats 1 and 2, a hydrophobic transmembrane domain, and a cytoplasmic tail. The locations of point mutations and small deletions in the major SARS-CoV-2 variant spike proteins are shown diagrammatically and on the crystal structure in Fig. 1B. Mutations of concern are those lying in the RBD, which is the binding site for most neutralizing antibodies, and those within the RBM (positions 453, 477, 484, and 501), which directly contacts ACE2. Two mutations lie near the processing site, and others are in the S1 N-terminal domain (NTD) and S2. Whether the mutations act independently or in a coordinated fashion is not known, and whether they were selected or are simply markers is not clear.

### Efficient pseudotyping of lentiviruses by variant spike proteins.

Lentiviral pseudotypes provide a rapid and accurate means to assess spike protein function. Neutralizing antibody titers determined by lentiviral pseudotype assays closely mirror those measured by live SARS-CoV-2 assays (38). To analyze the functional properties of the spike protein variants, we constructed cytomegalovirus (CMV) promoter-driven expression plasmids encoding the B.1.1.7, B.1.351, B.1.1.248, COH.20G/677H, 20A.EU2, and mink cluster 5 spike proteins or the component mutations, singly and in combination. Vector coding sequences were based on the Wuhan-Hu1 S gene with a deletion of the carboxy-terminal 19 amino acids that increases spike incorporation into virions. In this study, the D614G spike protein is considered the “wild type,” and the variants tested contain G614. The expression vectors were used to generate lentiviral pseudotypes with a packaged genomic RNA encoding both green fluorescent protein (GFP) and nanoluciferase. Immunoblot analysis showed that each spike protein was expressed in cells and incorporated into virions at levels comparable to those of the wild-type D614G spike protein, with the exception of T716I and the fully mutated B.1.1.7 spike proteins, which were expressed at lower levels (see Fig. S1A to C in the supplemental material). Because the B.1.1.7 spike protein with the full complement of mutations was poorly expressed, we used a triple mutant containing the critical Δ69–70/N501Y/P681H B.1.1.7 mutations. Measurement of spike protein proteolytic processing, as determined by the ratio of processed (S2) to full-length (S) proteins, showed that some of the mutations in the B.1.1.7 spike protein increased the extent of processing (N501Y, A570D, P681H, T716I, Δ69–70/N501Y, and Δ69–70/N501Y/P681H) (Fig. S1A). B.1.351, B.1.1.248, 20A.EU2, B.1.1.248, and COH.20G/677H were all processed to an extent similar to that of D614G (Fig. S1B and C).

10.1128/mBio.00696-21.1 FIG S1 Immunoblot analysis of spike protein expression and incorporation into lentiviral particles. Pseudotyped viruses were generated by transfection into 293T cells. On day 2, cells and supernatant virions were collected, lysed, and analyzed on an immunoblot probed with anti-spike antibody to detect the full-length spike protein and the S2 subunit. Cell lysates were probed with anti-GAPDH antibody to normalize for protein loading, and virions were probed for p24 to normalize for virions. Arrows indicate full-length spike (S), the S2 subunit (S2), p24, and GAPDH. (A) Immunoblot analysis of spike proteins with individual B.1.1.7 mutations. The histogram on the right shows the ratio of S2 to the full-length subunit determined by quantification of the bands. (B) Immunoblot analysis of spike proteins with individual B.1.351 mutations. The histogram on the right shows the ratio of the S2 subunit to full-length spike protein determined by quantification of the bands. (C, left and middle) Immunoblot analysis of spike proteins with D614G, B.1.1.7, and B.1.351 mutations (left) and 20A.EU2, COH20G/677H, and B.1.1.248 spike proteins (middle). (Right) Histogram showing the ratio of the S2 subunit to the full-length spike protein determined by quantification of the bands. Download FIG S1, TIF file, 1.5 MB. Copyright © 2021 Tada et al. 2021 Tada et al. https://creativecommons.org/licenses/by/4.0/ This content is distributed under the terms of the Creative Commons Attribution 4.0 International license.

The infectivity of pseudotyped virus with each of the variant spike proteins was tested by infection of ACE2.293T cells, a cell line that expresses high levels of ACE2, with normalized amounts of pseudotyped viruses. Analysis of the B.1.1.7 variant and its component mutations showed that the single point mutations had little effect on infectivity (Δ69–70, Y144Del, N501Y, A570D, P681H, and D1118H), except for T716I, which had 5.8-fold decreased infectivity, and S982A, which had significantly increased (1.6-fold) infectivity (Fig. 1C). The double mutant Δ69–70/N501Y and the triple mutant Δ69–70/N501Y/P681H also had increased infectivity (1.3- and 1.5-fold), suggesting that the two mutations coordinate to increase infectivity. The COH.20G/677H and 20A.EU2 variant pseudotypes were fully infectious, while B.1.1.248 was about 10-fold less infectious (Fig. 1D). Analysis of the B.1.351 variant showed that the individual point mutations had similar infectivities (0.8- to 1.04-fold), while the full complement was slightly (1.3-fold) decreased (Fig. 1E). Pseudotyped viruses with the mink-associated mutations were fully infectious except for the spike protein containing all 4 mutations (Δ69–70/Y453F/I692V/M1229F), which was 2-fold reduced in infectivity (Fig. 1F).

To determine whether the differences in infectivity could be caused by the effects of the mutations on the stability of the spike proteins or their incorporation into virions, we analyzed the spike proteins produced in transfected cell lysates and incorporated into virions. The transfected cell lysates were analyzed on an immunoblot probed for the spike protein S2, which allows the detection of the full-length spike protein and processed S2 protein, and for the HIV-1 capsid protein p24 as a means of normalizing for particle number. Analysis of B.1.1.7 showed that each of the singly mutated proteins was expressed in cells at similar levels and processed to similar extents (Fig. S1A). In contrast, some of the point mutations appeared to affect the amount of spike on the virion and the extent of spike protein processing. T716I was expressed at a significantly lower level, accounting for the decreased infectivity of this spike protein. P681H was present at a high copy number but was processed more efficiently than the wild type, as demonstrated by a higher ratio of S2 to full-length protein levels. The N501Y and A570D mutations resulted in a small decrease in the copy number on virions. When combined with Δ69–70, processing returned to the wild-type level. The addition of the P681H mutation in the triple mutant increased processing to a level similar to that of the P681H single point mutation. Analysis of the variants containing the full complement of mutations showed that the B.1.351, B.1.1.248, 20A.EU2, and COH.20G/677H spike proteins were stable and processed like the parental D614G, while B.1.1.7 was poorly expressed and present at a low copy number on virions (Fig. S1C).

### Convalescent-phase sera neutralize B.1.1.7, B.1.351, and B.1.1.248 pseudotyped viruses.

**FIG 2:**
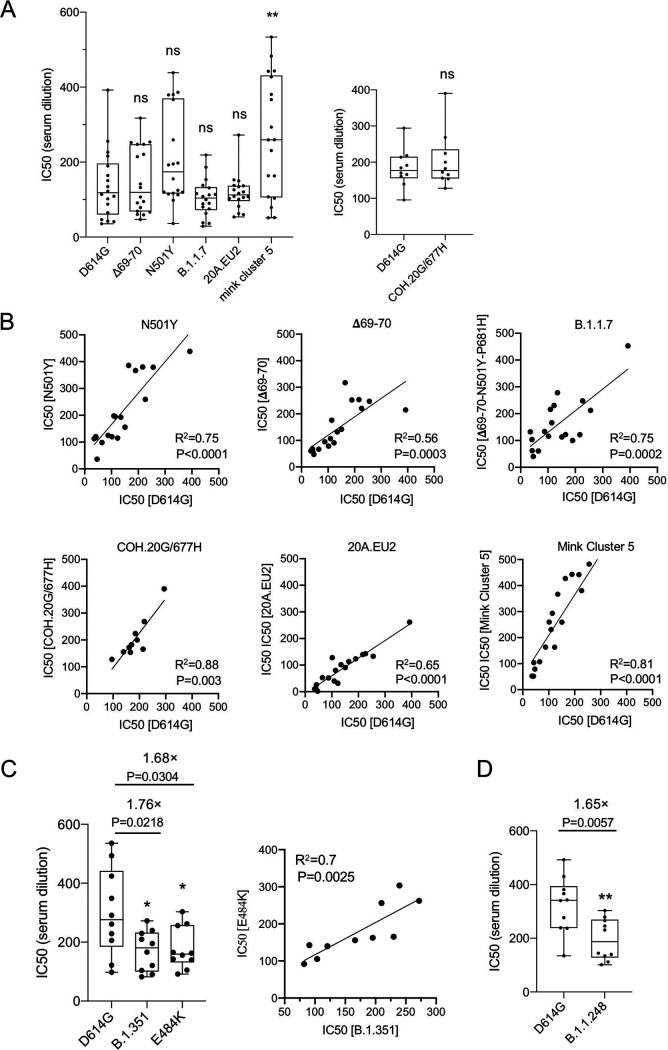
Neutralization of spike protein variants by convalescent-phase sera. (A) Neutralization of spike protein-pseudotyped viruses by serum samples from 18 (donors 1 to 18) (left) and 10 (donors 19 to 28) (right) convalescent individuals. The COH.20G/677H variant was tested in a separate experiment, as shown on the right. Each dot represents the IC_50_ for a single donor. Correlations were calculated with GraphPad Prism software using Pearson’s correlation coefficients, and error bars indicate standard deviations. The analyses were repeated twice, with similar results. n.s., not significant. (B) Neutralization by convalescent-phase donor serum of viruses pseudotyped by N501Y, Δ69–70, B.1.1.7 (Δ69–70/N501Y/P681H), COH.20G/677H, 20A.EU2, and mink cluster 5 spike proteins compared to D614G. The IC_50_s are based on serum dilution. (C, left) Neutralization of virus pseudotyped by the D614G, B.1.351, or E484K spike proteins by convalescent-phase sera. (Right) Comparison of neutralization of B.1.351 spike protein-pseudotyped virus with neutralization of the E484K pseudotype. (D) Neutralization of virus pseudotyped by the D614G and B.1.1.248 spike proteins by convalescent-phase sera.

A factor in determining how well recovered patients are protected from reinfection with spike protein variant viruses is the extent to which antibodies elicited in primary infection cross-react with emerging viral variants. To determine the extent of antibody cross-reactivity, we tested neutralizing antibody titers of the serum specimens from convalescent patients who had been infected prior to April 2020, a period prior to the emergence of the variants, for neutralizing titers against pseudotypes with variant spike proteins. Virus pseudotyped by the B.1.1.7 spike protein was neutralized slightly (1.3-fold) less well than the parental D614G, an effect that was noticeable in the lack of donors with high neutralizing titers. Pseudotypes with spike proteins bearing the single mutations (Δ69–70 and N501Y) were neutralized as efficiently as the parental D614G (Fig. 2A, left). Pseudotypes with the 20A.EU2 spike and the COH.20G/677H spike were neutralized with titers similar (0.9- and 1.1-fold, respectively) to that of D614G (Fig. 2A, right, and Tables 1 and 2). A direct comparison of neutralizing titers of B.1.1.7 (Δ69–70, N501Y, and Δ69–70/N501Y/P681H) with those of D614G showed a close correlation of neutralizing titers for each donor (Fig. 2A and B and Tables 1 and 2). This was also the case for COH.20G/677H, 20A.EU2, and mink cluster 5 spike variants, which were more easily neutralized (1.9-fold) than the D614G pseudotype. A detailed analysis of two donor sera chosen at random showed that the sera neutralized B.1.1.7 and its constitutive point mutations similarly, with the exception of T716I, which was more easily neutralized than D614G (Fig. S2).

**TABLE 1:** IC_50_s of convalescent-phase sera against D614G, Δ69–70, N501Y, B.1.1.7, 20A.EU2, and mink cluster 5 viruses with variant spike proteins

Donor	Serum IC_50_ (1/dilution)					
	D614G	Δ69–70	N501Y	B.1.1.7	20A.EU2	Mink cluster 5
1	255.8	247.4	379.5	219.0	152.7	482.8
2	226.6	247.4	259.5	186.3	150.0	380.7
3	150.8	220.5	155.5	88.74	103.2	259.3
4	64.9	142.0	98.19	38.23	60.02	107.1
5	42.42	66.92	116.9	36.42	53.93	103.9
6	87.77	59.4	124.9	138.2	62.72	163.7
7	46.43	94.81	36.21	28.98	137.9	78.92
8	109.0	47.15	197.7	63.45	100.9	230.9
9	122.6	106.9	114.9	75.12	108.7	163.3
10	134.8	91.06	192.5	80.2	105.8	366.7
11	102.0	132.0	119.8	131.3	134.5	259.7
12	392.3	78.69	438.4	153.5	272.4	533.5
13	189.7	214.7	366.7	115.9	126.1	443.1
14	216.4	252.4	380.0	112.7	136.6	442.1
15	164.1	253.7	386.0	90.07	120.6	427.2
16	41.3	317.4	120.0	113.8	107.5	51.63
17	35.43	69.1	112.6	108.5	116.6	52.32
18	113.9	60.66	195.3	99.85	82.18	293.4

Avg (SD)	138.7 (92)	150.1 (88)	210.8 (125)	104.5 (50)	118.5 (48)	268.9 (159)

*P* value (vs D614G)		0.71	0.57	0.18	0.42	0.005

**TABLE 2:** IC_50_s of convalescent-phase sera against D614G and COH.20G/677H viruses with variant spike proteins

Donor	Serum IC_50_ (1/dilution)	
	D614G	COH.20G/677H
19	218.3	268.5
20	95.56	127.9
21	184.3	224.1
22	170.2	182.4
23	139.4	155.7
24	213.8	165.6
25	161.2	172.1
26	165.8	153.9
27	191.2	199.8
28	294.2	390.3

Avg (SD)	183.4 (53)	204.0 (77)

*P* value (vs D614G)		0.49

10.1128/mBio.00696-21.2 FIG S2 Viruses pseudotyped by B.1.1.7 are neutralized by convalescent-phase serum. Convalescent-phase serum neutralization titration curves of pseudotyped viruses with individual B.1.1.7 mutations are shown. The experiments were repeated twice, with similar results. Download FIG S2, TIF file, 0.9 MB. Copyright © 2021 Tada et al. 2021 Tada et al. https://creativecommons.org/licenses/by/4.0/ This content is distributed under the terms of the Creative Commons Attribution 4.0 International license.

Analysis of B.1.351 and its constituent E484K point mutation showed that both viruses were neutralized by convalescent-phase sera with titers similar to that of D614G (Fig. 2C and Fig. S3A and B). For some donors, the neutralization curves were virtually identical (donors 1, 4, 5, 7, and 8), and for others, the variant spike protein pseudotypes were neutralized with slight reduction in titers (donors 2, 3, 6, 9, and 10). Overall, the reduction in the 50% inhibitory concentrations (IC_50_s) of B.1.351, B.1.1.248, and E484K pseudotypes was about 1.7-fold (Fig. 2C and D). The IC_50_ for each donor for the E484K single mutant was similar to that of B.1.351, suggesting that the E484K single amino acid change was responsible for the decrease in neutralizing titer (Fig. 2C). Spike proteins containing each of the other individual B.1.351 mutations were neutralized as well as D614G (Fig. S3C).

10.1128/mBio.00696-21.3 FIG S3 Convalescent-phase serum neutralization titration curves of pseudotyped viruses with B.1.351 mutations and individual mutations. (A) Neutralization of virus with the South Africa B.1.351 pseudotyped virus by sera of 10 convalescent individuals. The data represent percent infectivity not neutralized by convalescent-phase serum. (B) Serum neutralization curves of virus pseudotyped by the E484K spike protein. (C) Neutralization of viruses pseudotyped by individual B.1.351 mutations by sera of 5 convalescent individuals. (Top) Percent infectivity not neutralized by convalescent-phase sera. (Bottom) Neutralization IC_50_s of viruses pseudotyped by the D614G and individual B.1.351 mutations by convalescent-phase sera. The experiments were repeated twice, with similar results. Download FIG S3, TIF file, 1.5 MB. Copyright © 2021 Tada et al. 2021 Tada et al. https://creativecommons.org/licenses/by/4.0/ This content is distributed under the terms of the Creative Commons Attribution 4.0 International license.

### Antibodies elicited by BNT162b2 vaccination neutralize B.1.1.7, B.1.351, B.1.1.248, 20A.EU2, and COH.20G/677H pseudotyped viruses.

**FIG 3:**
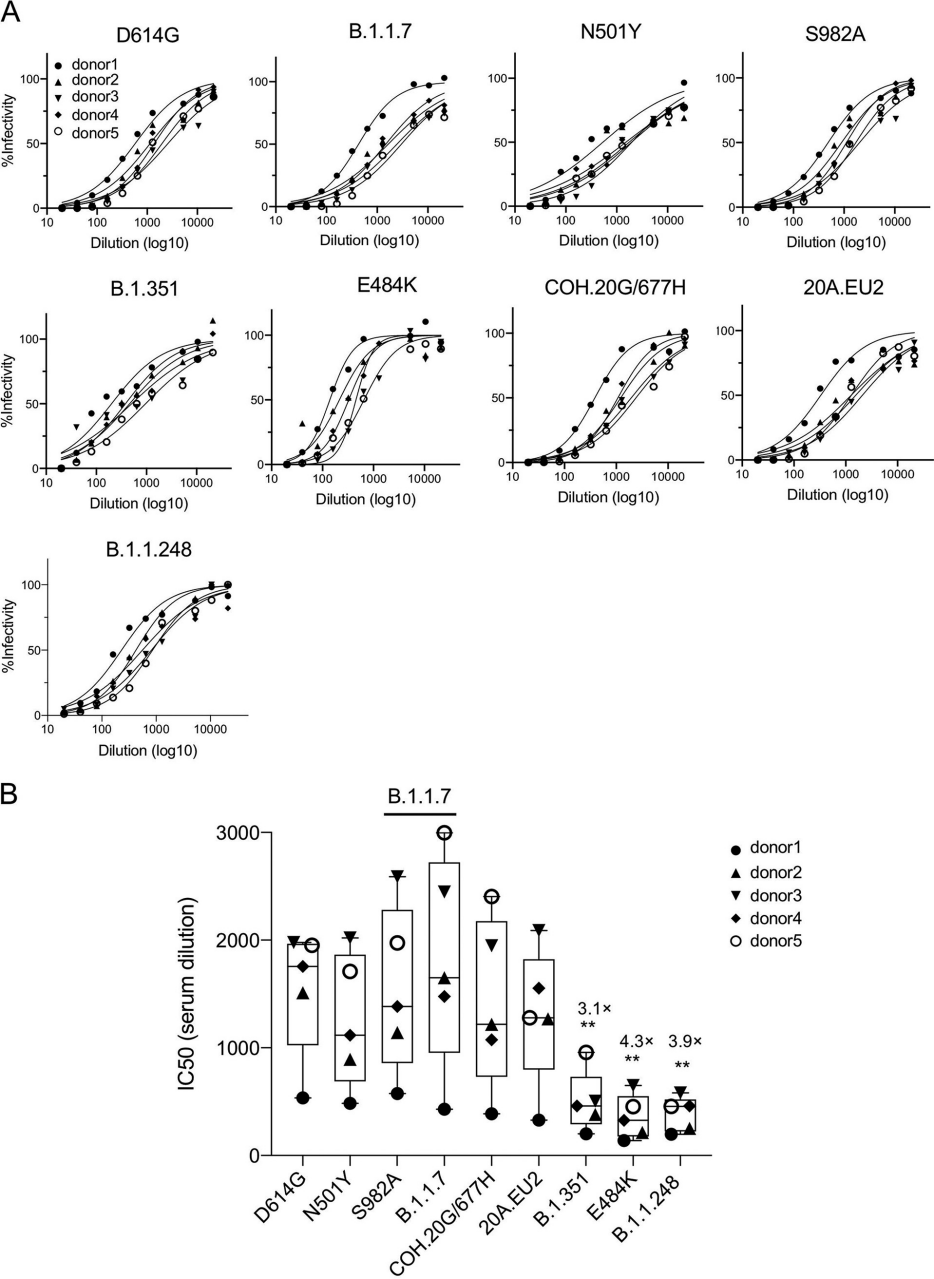
Neutralization of variant spike protein pseudotypes by the sera of BNT162b2-vaccinated individuals. (A) Serum samples from vaccinated individuals (*n* = 5) were serially diluted and incubated with the indicated variant spike protein-pseudotyped viruses normalized for infectivity. The data are relative to the infectivity of unneutralized virus. (B) Neutralization IC_50_s of D614G, N501Y, S982A, B.1.1.7 (Δ69–70/N501Y/P681H), COH.20G/677H, 20A.EU2, B.1.351, and B.1.1.248 pseudotypes. The experiments were repeated twice, with similar results.

The efficacy of current SARS-CoV-2 vaccines, which are based on spike proteins present prior to the emergence of viral variants, will be affected by how well the vaccine-elicited antibodies cross-react on circulating viral variants. To address this question, we analyzed the neutralizing activity of serum specimens from individuals vaccinated with the Pfizer mRNA vaccine against viruses pseudotyped by the B.1.1.7, B.1.351, and B.1.1.248 spike proteins. Sera were collected from five healthy donors on days 0, 7, and 28, where day 28 corresponded to 7 days after the second vaccine injection. At days 0 and 7, no detectable neutralizing antibody was detected, indicating that the donors had not been previously infected (not shown). On day 28, all donors had high titers of neutralizing antibodies against virus with the D614G spike protein, with an average neutralizing titer of 1:1,800, 7-fold higher than that of convalescent-phase serum samples (Fig. 3A and B). Neutralization titers against N501Y, S982A, B.1.1.7 (Δ69–70/N501Y/P681H), COH.20G/677H, and 20A.EU2 were similar to that against D614G (Fig. 3A and B), while B.1.351, B.1.1.248, and E484K were neutralized with 3.1-, 2.7-, and 4.3-fold decreases in titers. While this is a significant drop in titers, it remains higher than the titer found for convalescent-phase sera against D614G pseudotyped virus. The decrease in the neutralization of B.1.351 and B.1.1.248 appears largely due to the E484K mutation (Fig. 3A and B).

### Decreased neutralization of SARS-CoV-2 global variants by therapeutic anti-spike protein monoclonal antibodies.

**FIG 4:**
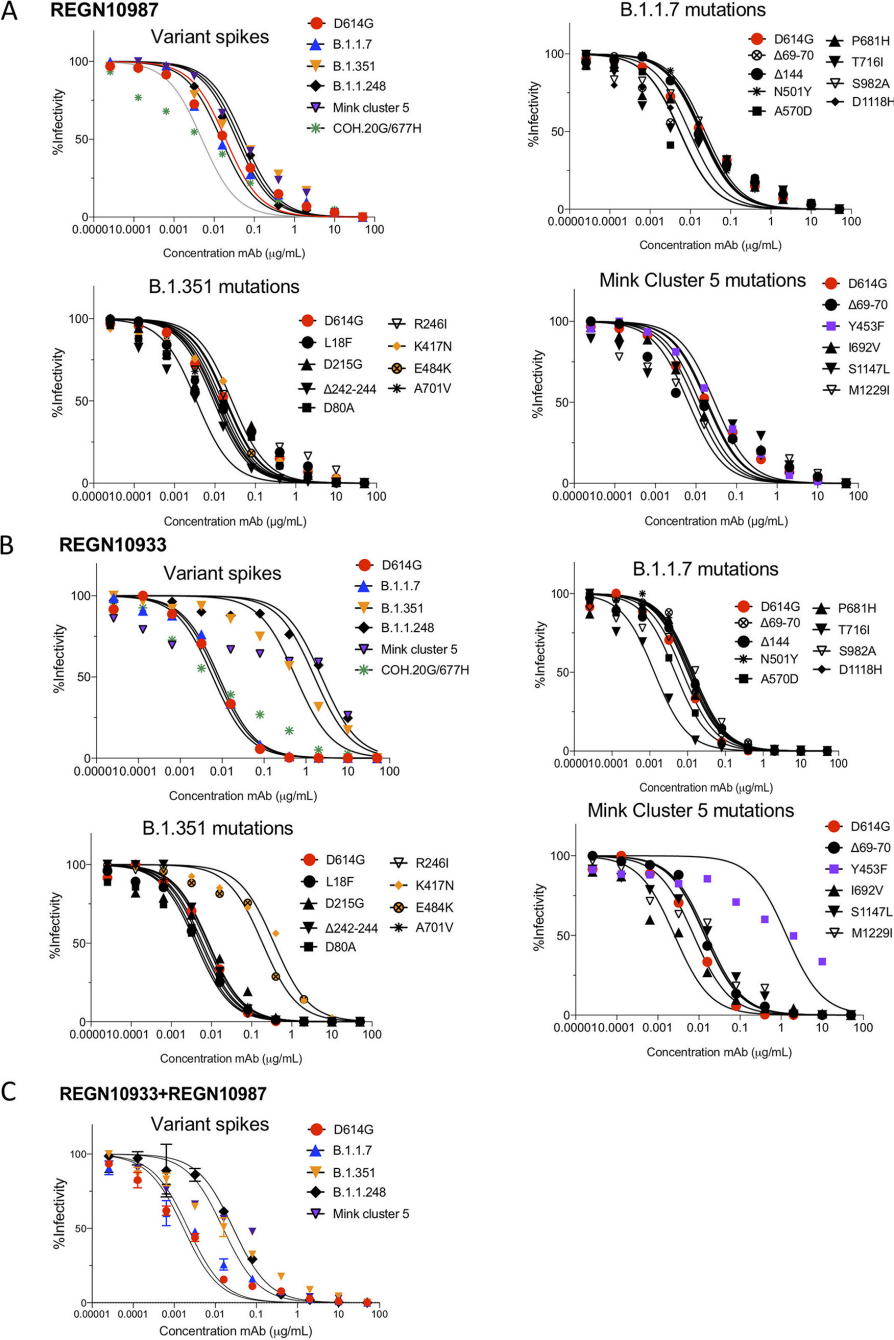
Neutralization of variant spike protein pseudotypes by REGN10933 and REGN10987 monoclonal antibodies. The neutralization of D614G, B.1.1.7, B.1.351, B.1.1.248, and mink cluster 5 pseudotyped viruses by REGN10933 and REGN10987 was measured. (A) Neutralization of B.1.1.7 (Δ69–70/N501Y/P681H), B.1.351, B.1.1.248, mink cluster 5, and COH.20G/677H pseudotypes (top left); individual B.1.1.7 mutations (top right); individual B.1.351 mutations (bottom left); and mink cluster 5 mutation pseudotyped viruses (bottom right) by REGN10987. (B) Neutralization curves of D614G, B.1.1.7 (Δ69–70/N501Y/P681H), B.1.351, B.1.1.248, mink cluster 5, and COH.20G/677H (top left); individual B.1.1.7 mutations (top right); individual B.1.351 mutations (bottom left); and mink cluster 5 mutation pseudotyped viruses (bottom right) by REGN10933. (C) Neutralization of D614G, B.1.1.7 (Δ69–70/N501Y/P681H), B.1.351, B.1.1.248, COH.20G/677H, and mink cluster 5 pseudotyped viruses by a 1:1 mixture of REGN10933 and REGN10987. The analyses were repeated three times, with similar results.

Because monoclonal antibodies bind to specific epitopes in the spike protein, they are particularly susceptible to escape by variant spike proteins. To determine whether the monoclonal antibodies constituting REGN-COV2 therapy are active against the variants, we tested the neutralizing activities of REGN10933 and REGN10987. We found that REGN10987 neutralized D614G with an IC_50_ of 19.4 ng/ml (Fig. 4A and Table 3) and neutralized B.1.1.7 and COH.20G/677H with similar titers, while B.1.351 and mink cluster 5 spike proteins were neutralized with slightly higher IC_50_s (2.2-fold and 2.8-fold, respectively). Viruses pseudotyped by the individual B.1.1.7 mutations were similarly neutralized, as were those of B.1.351 and mink cluster 5. REGN10933 was highly active against D614G, B.1.1.7, and COH.20G/677H, with IC_50_s of 7.4, 8.4, and 6.0 ng/ml, respectively, but had weak activity against B.1.351, B.1.1.248, and mink cluster 5, with IC_50_s 76.3-, >260-, and 214.9-fold higher, respectively, than that of D614G (Fig. 4B and Table 3). Analysis of the single mutations of B.1.351 showed that the escape from REGN10933 was due to K417N and E484K, each of which on its own was sufficient. The escape from the mink cluster 5 variant was caused by Y453F (Fig. 4B and Table 3). The combination of REGN10933 and REGN10987 was highly potent against virus with the D614G spike, with an IC_50_ of 1.69 ng/ml, but against B.1.351, B.1.1.248, and mink cluster 5, neutralizing titers were decreased 9.14-, 15.7-, and 16.2-fold, respectively, compared to D614G. The decrease in the neutralizing titer of the combination resulted in a major effect on REGN10933 combined with a minor effect on REGN10987 neutralization (Fig. 4C and Table 3). Analysis of the single point mutations showed that the decrease in neutralizing titers was caused by E484K, K417N, and Y453F mutations (Fig. 4 and Table 3).

**TABLE 3:** IC_50_s of recombinant monoclonal antibodies against viruses with variant spike proteins

Virus variant	IC_50_ (ng/ml)^*a*^		
	REGN10933	REGN10987	REGN10933 + REGN10987
D614G	7.44	19.42	1.69

B.1.1.7			
Δ69–70/N501Y/P681H	8.40	14.94	2.18
Δ69–70	14.03	9.49	
Δ144	11.11	19.79	
N501Y	12.22	18.40	
A570D	4.68	5.52	
P681H	10.16	17.47	
T716I	1.32	5.41	
S982A	12.92	23.00	
D1118H	10.17	17.56	

B.1.351	567.60	43.74	15.45
L18F	4.51	9.28	
D80A	4.02	11.46	
D215G	8.56	12.85	
Δ242–244	8.26	3.48	
R246I	6.04	18.27	
K417N	362.60	26.17	
E484K	54.29	10.55	
N501Y	12.22	18.45	
A701V	5.43	14.98	

B.1.1.248	>2,000	36.37	26.66

Mink cluster 5	1,599.0	54.33	27.44
Δ69–70	14.03	9.49	
Y453F	1,355.0	28.81	
I692V	2.54	12.40	
S1147L	15.44	17.99	
M1229I	13.72	6.49	

COH.20G/677H	5.98	4.96	

F486S	>2,000	8.49	8.05

a Shading indicates escape mutants.

### Variant spike proteins have a higher affinity for ACE2 and increased thermostability.

**FIG 5:**
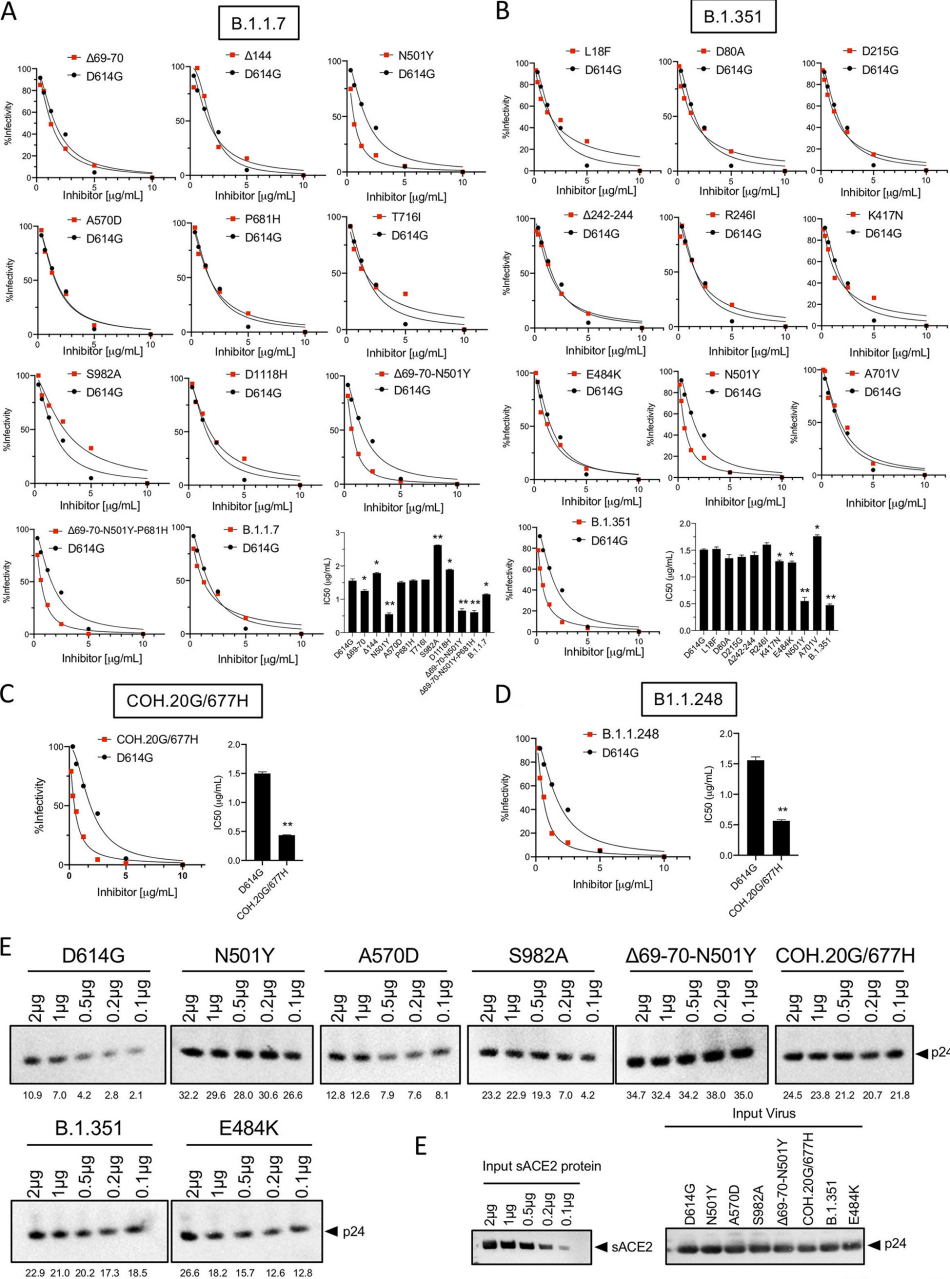
Relative affinity of viruses pseudotyped by B.1.1.7 and B.1.351 spike proteins for ACE2. The relative affinity of the variant spike proteins for ACE2 was measured by a soluble ACE2 (sACE2) neutralization assay (A to D) and a virion binding assay (E). For the sACE2 neutralization assay, viruses pseudotyped with variant spike proteins were incubated with a serial dilution of recombinant sACE2, and their infectivity was then measured on ACE2.293T cells. The data represent percent infectivities, at each concentration of sACE2, of the spike variants plotted against sACE2 neutralization of D614G pseudotyped virus. The histograms at the bottom right summarize the IC_50_s for each of the curves. For the virion binding assay, pseudotyped virions (30 ng p24) were incubated with Ni-NTA beads coated with the indicated amounts of His-tagged recombinant sACE2 protein. Unbound virions were removed by centrifugation, and bound virions were analyzed on an immunoblot probed with anti-p24 antibody. (A) sACE2 neutralization of viruses pseudotyped by spike proteins with the individual B.1.1.7 mutations. (B) sACE2 neutralization of viruses pseudotyped by spike proteins with the individual B.1.351 mutations. (C) sACE2 neutralization of COH.20G/677H (left) and B.1.1.248 (right) pseudotyped viruses. The histograms to the right of the curves show the calculated IC_50_s. (D) Virion binding assay of viruses pseudotyped with the variant spike proteins. (E) Immunoblot analysis of the input sACE2 (left) and pseudotyped viruses (right) used in the virus-sACE2 binding assay. The mass of bead-bound p24 (nanograms) as determined using a standard curve with recombinant protein is indicated below each lane.

The D614G mutation caused significant increases in viral infectivity and binding to ACE2 (2, 39, 40). We previously reported increased binding using an *in vitro* assay in which spike protein-pseudotyped virus was incubated with beads coated with soluble ACE2 (sACE2) (41). We have now developed a more sensitive assay that is based on the neutralization of pseudotyped virus by sACE2 in solution. In this assay, a higher affinity for ACE2 binding results in increased sensitivity to sACE2 neutralization. In this analysis, the B.1.1.7 pseudotyped virus did not show increased ACE2 affinity compared to D614G. In contrast, pseudotyped virus containing the single N501Y mutation, as well as those that included N501Y (Δ69–70/N501Y and Δ69–70/N501Y/P681H), showed increased ACE2 binding (Fig. 5A). B.1.351 also showed increased ACE2 binding, and this was due to N501Y, as none of the other point mutations had an effect (Fig. 5B). COH.20G/677H and B.1.1.248 pseudotyped viruses, which also contain N501Y, also displayed increased ACE2 binding (Fig. 5C). Analysis of the variant spike proteins in an ACE2 binding assay in which virions were incubated with matrix-bound sACE2 confirmed the finding that spike proteins that contained N501Y had an increased affinity for ACE2 (N501Y, Δ69–70/N501Y, COH.20G/677H, and B.1.351) (Fig. 5D).

The transmissibility of the viruses is likely to be affected by their stability in aerosols and resistance to high temperatures. To determine the stability of the variant spike proteins, pseudotyped viruses were incubated for 30 and 60 min at an elevated temperature. Incubation of the viruses for 1 h at 50°C caused a 40-fold decrease in the infectivity of the D614G virus (Fig. S4). Pseudotyped viruses with N501Y, S982A, and Δ69–70/N501Y/P681H spikes and B.1.351 decreased their infectivity <20-fold, suggesting that the mutations increase spike protein stability.

10.1128/mBio.00696-21.4 FIG S4 Thermostability of variant spike protein-pseudotyped viruses. Viruses pseudotyped by the indicated spike proteins were incubated at 4°C, 37°C, 42°C, and 50°C for 30 min and 1 h, after which infectivity was determined in ACE2.293T cells. The experiments were repeated twice, with similar results. Download FIG S4, TIF file, 0.9 MB. Copyright © 2021 Tada et al. 2021 Tada et al. https://creativecommons.org/licenses/by/4.0/ This content is distributed under the terms of the Creative Commons Attribution 4.0 International license.

## DISCUSSION

Convalescent-phase sera from individuals who had been infected prior to the emergence of the variants neutralized viruses with the B.1.1.7, COH.20G/677H, 20A.EU2, and mink cluster 5 spikes with titers close to that of the parental D614G and neutralized viruses pseudotyped with B.1.351 and B.1.1.248 spike proteins nearly as well, with a 1.7-fold decrease in titer. The BNT162b2 mRNA vaccine elicited antibodies with an average 7-fold-higher titer than those elicited by natural infection. The vaccine-elicited antibodies neutralized virus pseudotyped with B.1.1.7, COH.20G/677H, and 20A.EU2 spike proteins with titers similar to that of D614G and neutralized the B.1.351 and B.1.1.248 spike proteins with a 3-fold decrease in titer, a titer that remained higher than that elicited by natural infection against D614G. The decrease in the neutralizing titer was attributable to the E484K mutation. Viruses pseudotyped with B.1.1.7 or B.1.351 were not more infectious than the parental D614G, although in an ACE2 binding assay, the N501Y mutation present in the B.1.1.7, B.1.351, and COH.20G/677H spike proteins caused an increase in the ACE2 binding affinity. Our findings are consistent with those in a recent report by Wu et al., who found no significant decrease in neutralizing titer for B.1.1.7 and a 6.4-fold decrease for virus with the B.1.351 spike protein by antibodies elicited by the Moderna mRNA-1273 vaccine (24). In addition, Xie et al. found that BNT162b2 vaccine-elicited antibodies neutralized virus with E484K/N501Y mutations with a titer that was 0.8-fold lower compared to D614G (23). Wang et al. found a more pronounced 9.4-fold decrease in the neutralization of B.1.351 by convalescent-phase serum and a 0.3- to 12.4-fold decrease for vaccine sera (25).

The variant spike proteins were expressed well in cells and efficiently incorporated into virions, with the exception of the B.1.1.7 spike protein and a spike protein with one of the constituent B.1.1.7 mutations, T716I. The mutation, which is located close to the fusion peptide, decreased infectivity 7-fold and decreased spike protein incorporation into virions with an 18-fold decrease in infectivity that was not restored by keeping amino acid 716 as threonine (not shown). The decreased infectivity of this pseudotype may be caused by a biosynthesis problem in 293T cells and probably does not reflect the situation *in vivo* where the B.1.1.7 virus is highly infectious.

The selective pressures driving the evolution of the spike protein are likely to be a combination of selection for increased infectivity and for escape from humoral and cellular immune responses. Increased infectivity could result from an improved affinity for ACE2, altered proteolytic processing of the spike protein, and increased stability of the virus in aerosols. Mutations driven by selection for increased infectivity could result in evasion of the humoral response. In our experiments, D614G caused a significant increase in infectivity (41), but the variants, all of which contain the mutation, showed either no increase or a small increase (1.1- to 1.6-fold) in infectivity (Δ69–70, S982A, and Δ69–70/N501Y/P681H). While the effects of the mutations on infectivity were minor, tissue culture does not model factors that pertain *in vivo*, such as thermostability or stability in aerosols. In an attempt to detect an effect on infectivity, we tested the thermostability of viruses pseudotyped by the variant spike proteins after 30 and 60 min. We found that the B.1.351, B.1.1.7, and some of the variant point mutations caused a >2-fold increase in infectivity at 50°C compared to D614G, suggesting that the mutations increase the stability of the spike protein (see Fig. S4 in the supplemental material). It will be of interest to further investigate the stability of viruses with the variant spike proteins under conditions that mimic real life to understand the basis of the increased transmissibility and prevalence of SARS-CoV-2 variants.

In this study, we used spike protein-pseudotyped lentiviruses to determine viral infectivity and antibody neutralization. While the viruses are not identical to native coronavirus virions, they provide an accurate measure of viral infectivity and antibody neutralization and provide a means to rapidly test variant spike proteins (42–47). A comparison of neutralizing titers measured on a panel of 101 convalescent-phase sera by lentiviral pseudotypes and live SARS-CoV-2 showed a high degree of concordance between the two assays (38). This is the case even though the two assays are based on viral particles that may not have similar numbers of spike protein trimers per virion. The assay does not measure potential beneficial antiviral effects of nonneutralizing antibody, which also may be induced by vaccination. It also does not measure T cell responses against viral proteins. Such responses may contribute to the protective effect of vaccination and would serve to further provide protection against infection with SARS-CoV-2 variants.

The neutralizing titer of antibodies elicited by BNT162b2 vaccination against virus pseudotyped by B.1.1.7, COH.20G/677H, and mink cluster 5 spike proteins was only modestly decreased. Vaccine-elicited antibodies neutralized B.1.351 and B.1.1.248 with a 3-fold decrease in titers, but given the 7-fold increase in neutralizing titers of antibodies elicited by vaccination compared to natural infection, it is likely that the vaccine will maintain a high degree of protection. It was the case that one donor serum sample tested had a relatively low titer against the variant spike proteins. Whether the low titer would result in decreased protection will require a better understanding of the correlates of protection. The decrease in titer was the result of the E484K mutation, suggesting that a modified vaccine with this mutation might be required to provide a high degree of protection for such individuals.

Our analysis of the REGN-COV2 therapeutic monoclonal antibodies showed that REGN10987 maintained most of its activity against the variant spike proteins and that REGN10933 lost most of its neutralizing activity against the B.1.351, B.1.1.248, and mink cluster 5 variant spike proteins. The escape was the result of the K417N and E484K mutations in the RBD, either of which prevents neutralization, consistent with the findings of Wang et al. (25). The combined REGN10933 and REGN10987 cocktail had a 9.1-fold decrease in neutralizing titers against B.1.351, a 15.7-fold decrease against B.1.1.248, and a 16.2-fold decrease against mink cluster 5. REGN10933 and REGN10987 bind to nonoverlapping sites on the RBD (35). REGN10933 binds on the top of the RBD, blocking the interaction with ACE2, while REGN10987 binds to the side of the RBD and does not overlap the ACE2 binding site (35). The mutations that affected REGN10933 (E484K, K417N, and Y453F) cluster on the face of the RBD to which the antibody binds (Fig. 1B). In addition, neutralization by REGN10933 was prevented by the nearby mutation F486S, a mutation that has been shown to affect ACE2 binding (48) (Fig. S5 and Table 3). The sensitivity of REGN10933 to mutations in spike protein variants may result from selective pressure to increase the ACE2 binding affinity, a pressure that is not as great for amino acids located on the face of the RBD bound by REGN10987. Using a deep mutational scanning method, Starr et al. found that mutations at residue 486 escape neutralization by REGN10933, whereas mutations at residues 439 and 444 escape neutralization by REGN10987 (18). A single mutation, E406W, allowed escape from both antibodies, although the residue is not located within the epitope bound by either antibody. Analysis of spike protein mutations that occurred in a treated immunocompromised patient revealed additional mutations that allowed escape from either antibody (49). Whether such escape mutations will become clinically problematic is not clear as the mutations may decrease viral fitness and therefore not become frequent in the human population. Our findings highlight the benefit of a two-antibody cocktail as a single antibody could lose effectiveness in patients infected with viral variants. Whether the decreased neutralizing titer of the cocktail will translate into a loss of clinical effectiveness for individuals infected with the B.1.351 variant is not clear.

10.1128/mBio.00696-21.5 FIG S5 Neutralization curves for REGN10933 and REGN10987 on F486S spike protein-pseudotyped virus. Neutralization by REGN10933 and REGN10987 of lentiviral pseudotyped virions with F486S or D614G mutations in the spike protein was analyzed. F486S is not one of the major circulating variants. The experiments were repeated three times, with similar results. Download FIG S5, TIF file, 0.7 MB. Copyright © 2021 Tada et al. 2021 Tada et al. https://creativecommons.org/licenses/by/4.0/ This content is distributed under the terms of the Creative Commons Attribution 4.0 International license.

SARS-CoV-2 will likely continue to evolve, driven by selection for increased transmissibility and evasion of the host immune response. The results reported here highlight the importance of worldwide surveillance of circulating viruses by nucleotide sequencing and the need to monitor novel spike protein variants for neutralization by convalescent-phase and vaccine sera and therapeutic monoclonal antibodies. It will be important to define correlates of protection to determine whether there is a need to produce modified vaccines and to develop monoclonal antibodies that target highly conserved spike protein epitopes that the virus cannot readily mutate.

## MATERIALS AND METHODS

### Plasmids.

pLenti.GFP.NLuc is a dual GFP/nanoluciferase lentiviral vector based on pLenti.CMV.GFP.puro containing a GFP/nanoluciferase cassette separated by a picornavirus P2A self-processing amino acid motif cloned into the BamHI and SalI sites (Addgene plasmid 17448, provided by Eric Campeau and Paul Kaufman) (50). pcCOV2.Δ19S is based on pCDNA6 in which the CMV promoter drives the transcription of a synthetic, codon-optimized SARS-CoV-2 spike gene based on Wuhan-Hu1/2019 with a termination codon at position 1255 that deletes the carboxy-terminal 19 amino acids (41). Point mutations were introduced by overlap extension and confirmed by DNA nucleotide sequence analysis. The HIV-1 Gag/Pol expression vector pMDL and the HIV-1 Rev expression vector pRSV.Rev were previously described (41).

### Human sera and monoclonal antibodies.

Convalescent-phase sera and sera from individuals vaccinated at NYULH with BNT162b2 on day 0, day 7, and day 28 (7 days following the second injection) were collected from individuals through the NYU Vaccine Center with written consent under institutional review board (IRB) approval (IRB 20-00595 and IRB 18-02037) and were deidentified. Donor age and gender were not reported.

cDNAs encoding REGN10933 and REGN10987 were synthesized using published sequences; fused to the IgG1 heavy chain and lambda light chains, respectively; and cloned into pcDNA3.1 (Invitrogen). The proteins were produced in transfected Freestyle 293 cells and collected from the cell supernatant after 4 days. The antibodies were purified on an Äkta prime fast protein liquid chromatography (FPLC) system with HiTrap Pro A 5-ml column. The proteins were tested for purity by SDS-PAGE, quantified by a bicinchoninic acid (BCA) assay, and tested for spike protein binding by biolayer interferometry on an Octet detection system.

### Cells.

293T cells were cultured in Dulbecco’s modified Eagle medium (DMEM) supplemented with 10% fetal bovine serum (FBS) and penicillin/streptomycin (P/S) at 37°C in 5% CO_2_. ACE2.293T cells are clonal cell lines established by stable cotransfection with pLenti.ACE2-HA followed by selection in 1 μg/ml puromycin, as previously described (38, 41).

### SARS-CoV-2 spike lentiviral pseudotypes.

SARS-CoV-2 spike protein-pseudotyped lentiviral stocks were produced by the cotransfection of 293T cells with pMDL, pLenti.GFP-NLuc, pcCoV2.S-Δ19 (or variants thereof), and pRSV.Rev as previously described (41). Virus stocks were normalized by real-time PCR reverse transcriptase (RT) activity (51). Pseudotyped virus infections were done with 1 × 10^4^ cells/well in 96-well tissue culture dishes at a multiplicity of infection (MOI) of 0.2 as previously described (41). Luciferase activity was measured after 2 days using the Nano-Glo luciferase substrate (Promega), and plates were read in an Envision 2103 microplate luminometer (PerkinElmer). To quantify antibody neutralization, sera were serially diluted 2-fold and incubated for 30 min at room temperature with pseudotyped virus (corresponding to approximately 2.5 × 10^7^ cps luciferase) in a volume of 50 μl. The mixture was added to 1 × 10^4^ ACE2.293T cells (corresponding to an MOI of 0.2) in a volume of 50 μl in a 96-well culture dish. After 2 days, the medium was removed, and the Nano-Glo luciferase substrate (Nanolight) was added to wells. Luminescence was read in an Envision 2103 microplate luminometer (PerkinElmer).

### Immunoblot analysis.

Cells were lysed in buffer containing 50 mM HEPES, 150 mM KCl, 2 mM EDTA, 0.5% NP-40, and a protease inhibitor cocktail. Lysates (40 μg) were separated by SDS-PAGE and transferred to a polyvinylidene difluoride membrane. The membranes were probed with anti-spike protein mAb (1A9) (GeneTex), anti-p24 mAb (AG3.0), anti-His mAb (Invitrogen), and anti-glyceraldehyde-3-phosphate dehydrogenase (GAPDH) mAb (Life Technologies) followed by goat anti-mouse horseradish peroxidase (HRP)-conjugated second antibody (Sigma). The membrane was treated with a luminescent substrate (Millipore), and the band intensities were quantified on an iBright CL1000 imager.

### Virus ACE2 binding assay.

Soluble ACE2 containing a carboxy-terminal His tag (41) was serially diluted and mixed with 20 μl Ni-nitrilotriacetic acid (NTA) beads for 1 h at 4°C. Unbound protein was removed by washing with phosphate-buffered saline (PBS). The coated beads were mixed with 40 μl pseudotyped lentiviral virions and incubated for 1 h at 4°C. The beads were then washed with PBS, resuspended in reducing Laemmli loading buffer, heated to 90°C, and analyzed on an immunoblot probed with anti-HIV-1 p24 antibody AG3.0 followed by goat anti-mouse HRP-conjugated secondary antibody.

### Soluble ACE2 neutralization assay.

The soluble ACE2 neutralization assay has been previously described (41). Briefly, serially diluted recombinant soluble ACE2 protein was mixed with pseudotyped virus for 1 h at room temperature, added to 1 × 10^4^ ACE2.293T cells, and incubated for 2 days. After 2 days, the medium was removed, and 50 μl of the Nano-Glo luciferase substrate (Nanolight) was added. The luminescence was read in an Envision 2103 microplate luminometer (PerkinElmer).

### Quantification and statistical analysis.

All experiments were performed in technical duplicates or triplicates, and data were analyzed using GraphPad Prism 8. Statistical significance was determined by the two-tailed, unpaired *t* test. Correlation analysis was performed in GraphPad Prism 8 using Pearson’s correlation coefficients. Significance was based on two-sided testing and attributed to a *P* value of <0.05. Confidence intervals are shown as the means ± standard deviations (SD) or standard errors of the means (SEM) (*, *P* ≤ 0.05; **, *P* ≤ 0.01; ***, *P* ≤ 0.001; ****, *P* ≤ 0.0001). The PDB file for the D614G SARS-CoV-2 spike protein (accession number 7BNM) was downloaded from the Protein Data Bank. A three-dimensional (3D) view of the protein was obtained using PyMOL.
